# Pathway polygenic risk scores (pPRS) for the analysis of gene-environment interaction

**DOI:** 10.1371/journal.pgen.1011543

**Published:** 2025-08-05

**Authors:** W. James Gauderman, Yubo Fu, Bryan Queme, Eric Kawaguchi, Yinqiao Wang, John Morrison, Hermann Brenner, Andrew Chan, Stephen B. Gruber, Temitope Keku, Li Li, Victor Moreno, Andrew J. Pellatt, Ulrike Peters, N. Jewel Samadder, Stephanie L. Schmit, Cornelia M. Ulrich, Caroline Um, Anna Wu, Juan Pablo Lewinger, David A. Drew, Huaiyu Mi

**Affiliations:** 1 Division of Biostatistics and Health Data Science, Department of Population and Public Health Sciences, University of Southern California, Los Angeles, California, United States of America; 2 Division of Bioinformatics, Department of Population and Public Health Sciences, University of Southern California, Los Angeles, California, United States of America; 3 Division of Clinical Epidemiology and Aging Research, German Cancer Research Center (DKFZ), Heidelberg, Germany; 4 German Cancer Consortium (DKTK), German Cancer Research Center (DKFZ), Heidelberg, Germany; 5 Clinical and Translational Epidemiology Unit, Massachusetts General Hospital and Harvard Medical School, Boston, Massachusetts, United States of America; 6 Division of Gastroenterology, Massachusetts General Hospital and Harvard Medical School, Boston, Massachusetts, United States of America; 7 Center for Precision Medicine and Department of Medical Oncology, City of Hope National Medical Center, Duarte, California, United States of America; 8 University of North Carolina at Chapel Hill, Chapel Hill, North Carolina, United States of America; 9 Department of Family Medicine, UVA Comprehensive Cancer Center, UVA School of Medicine, Charlottesville, Virginia, United States of America; 10 Oncology Data Analytics Program, Catalan Institute of Oncology (ICO), L’Hospitalet de Llobregat, Barcelona, Spain; 11 Colorectal Cancer Group, ONCOBELL Program, Institut d’Investigació Biomèdica de Bellvitge (IDIBELL), L’Hospitalet de Llobregat, Barcelona, Spain; 12 Department of Clinical Sciences, Faculty of Medicine and Health Sciences and Universitat de Barcelona Institute of Complex Systems (UBICS), University of Barcelona (UB), L’Hospitalet de Llobregat, Barcelona, Spain; 13 Consortium for Biomedical Research in Epidemiology and Public Health (CIBERESP), Madrid, Spain; 14 Intermountain Health, Salt Lake City, Utah, United States of America; 15 Public Health Sciences Division, Fred Hutchinson Cancer Center, Seattle, Washington, United States of America; 16 Department of Epidemiology, School of Public Health, University of Washington, Seattle, Washington, United States of America; 17 Mayo Clinic Comprehensive Cancer Center, Phoenix, Arizona, United States of America; 18 Genomic Medicine Institute, Cleveland Clinic, Cleveland, Ohio, United States of America; 19 Population and Cancer Prevention Program, Case Comprehensive Cancer Center, Cleveland, Ohio, United States of America; 20 Huntsman Cancer Institute, Salt Lake City, Utah, United States of America; 21 Department of Population Sciences, University of Utah, Salt Lake City, Utah, United States of America; 22 Department of Population Science, American Cancer Society, Atlanta, GeorgiaUnited States of America; 23 Department of Population and Public Health Sciences, University of Southern California, Los Angeles, California, United States of America; University of Michigan, UNITED STATES OF AMERICA

## Abstract

A polygenic risk score (PRS) is used to quantify the combined disease risk of many genetic variants. For complex human traits there is interest in determining whether the PRS modifies, i.e. interacts with, important environmental (E) risk factors. Detection of a PRS by environment (PRS x E) interaction may provide clues to underlying biology and can be useful in developing targeted prevention strategies for modifiable risk factors. The standard PRS may include a subset of variants that interact with E but a much larger subset of variants that affect disease without regard to E. This latter subset will dilute the underlying signal in former subset, leading to reduced power to detect PRS x E interaction. We explore the use of pathway-defined PRS (pPRS) scores, using state of the art tools to annotate subsets of variants to genomic pathways. We demonstrate via simulation that testing targeted pPRS x E interaction can yield substantially greater power than testing overall PRS x E interaction. We also analyze a large study (N = 78,253) of colorectal cancer (CRC) where E = non-steroidal anti-inflammatory drugs (NSAIDs), a well-established protective exposure. While no evidence of overall PRS x NSAIDs interaction (p = 0.41) is observed, a significant pPRS x NSAIDs interaction (p = 0.0003) is identified based on SNPs within the TGF-β/ gonadotropin releasing hormone receptor (GRHR) pathway. NSAIDS is protective (OR=0.84) for those at the 5^th^ percentile of the TGF-β/GRHR pPRS (low genetic risk, OR), but significantly more protective (OR=0.70) for those at the 95^th^ percentile (high genetic risk). From a biological perspective, this suggests that NSAIDs may act to reduce CRC risk specifically through genes in these pathways. From a population health perspective, our result suggests that focusing on genes within these pathways may be effective at identifying those for whom NSAIDs-based CRC-prevention efforts may be most effective.

## Author summary

The identification of polygenic risk score (PRS) by environment (PRSxE) interactions may provide clues to underlying biology and facilitate targeted disease prevention strategies. The standard approach to computing a PRS likely includes many variants that affect disease without regard to E, reducing power to detect PRS x E interactions. We utilize gene annotation tools to develop pathway-based PRS (pPRS) scores and show by simulation studies that testing pPRS x E interaction can yield substantially greater power than testing PRS x E, while also integrating biological knowledge into the analysis. We apply our method to a large study of colorectal cancer to identify a significant pPRS x NSAIDs interaction (p = 0.0003) based on SNPs within the TGF-β/ gonadotropin releasing hormone receptor (GRHR) pathway. Our findings suggest that focusing on genetic susceptibility within biologically informed pathways may be more sensitive for identifying exposures that can be considered as part of a precision prevention approach.

## Introduction

Gene-environment (GxE) interactions likely play an important role in the etiology of most complex human traits [1]. A GxE analysis aims to identify genetically defined subsets of the population that may be more sensitive to adverse or protective effects of an exposure on disease risk. Alternatively, one can view G x E interaction as investigating whether a particular exposure stimulates or suppresses the effect of a gene on disease risk. The power to detect GxE interactions, particularly in the context of a genomewide scan, is lower than the power to detect similarly-sized genetic or environmental main effects [2]. Identification of actionable GxE interactions is essential to precision medicine approaches that are expected to transform the future of medicine, particularly for primary prevention of diseases.

A polygenic risk score (PRS) is commonly used to summarize the overall effect of a collection of identified genetic variants on a particular trait. The variants used to construct the PRS can be focused on a relatively small set identified by a prior GWAS or a much larger set that captures genome-wide genetic variation. The PRS can be used to characterize the total trait variance attributable to discovered variants or to identify specific subsets of the population likely to be at highest risk for disease [3,4].

Recently, many investigators have utilized PRS x E analysis to study gene-environment interactions for a wide range of traits, including lung cancer [5], diabetes [6], ADHD [7], and cardiovascular disease [8]. Compared to single-variant GxE analysis, PRS x E analysis may provide increased power because it focuses on known disease-related variants and it integrates the signals across those variants into a potentially more informative single measure of genetic susceptibility [9]. Detecting a PRS x E interaction will allow us to answer questions such as: Does the effect of a particular exposure on disease risk vary depending on overall genetic susceptibility? Do we need to consider specific exposures when making PRS-based risk predictions? Is there a particularly high-risk subgroup, defined by both genetic susceptibility and exposure, for whom targeted prevention (e.g. early screening) may be indicated?

Despite these advantages, a potential difficulty in identifying PRS x E is that standard construction of the PRS includes all GWAS-significant variants or a very large set of genomewide variants. Environmental factors likely work to affect disease risk by altering the functioning or expression of genes within specific pathways. Examples include smoking affecting DNA repair pathways to alter lung cancer risk [10] and red meat affecting inflammatory response pathways to affect colorectal cancer risk [11]. While a standard PRS may include several variants within an exposure-relevant pathway, its standard construction will tend to ‘water down’ the specific signals most important for identifying the interaction(s).

To overcome this challenge, we propose the use of pathway polygenic risk scores (pPRS) in gene-environment interaction analyses. Relative to a PRS, a pPRS may include a greater proportion of disease-related SNPs that individually or in combination interact with a particular exposure, and which in turn should provide greater power for detecting pPRS x E compared to PRS x E. We will describe the use of available functional annotation databases to define subsets of PRS SNPs according to their known pathway affiliation. Multiple pPRS can be constructed, each corresponding to a particular pathway and utilizing a subset of the overall collection of PRS SNPs. The use of pathway-specific PRS has been described for classifying disease subtypes [12–14] and enhancing drug target discovery [15], but to our knowledge not for identifying pPRS x E interactions. To illustrate our approach, we analyze PRS x E and pPRS x E interactions in a large study of colorectal cancer, focusing on over 200 GWAS-identified SNPs and a well-established protective exposure, non-steroidal anti-inflammatory drug (NSAID) use.

## Results

### Simulations

We designed a simulation study to determine whether power to detect pPRS x E interaction may be higher than for PRS x E interaction, and if so, under what conditions one may expect greater power. Briefly, we simulated 1,000 SNPs, of which 20 were assumed to affect disease (D) risk and 980 to have no effect on D. We also simulated a binary exposure (E) and generated 5 of the 20 SNPs to also have a GxE effect on D. We assumed 5 of the 1,000 SNPs fell within a pathway and varied how many of those 5 pathway SNPs overlapped with the 5 GxE SNPs, the 15 other disease-causing SNPs, and the remaining 980 null SNPs. We replicated the simulation 1,000 times and estimated power based on the proportion of replicates in which we detected interaction based on analysis of PRS x E vs. pPRS x E. Additional details of the simulation design, as well as demonstration that Type I error is preserved, are provided in Materials and Methods.

Across a wide range of simulated scenarios, power to detect interaction is greater for pPRSxE than for PRSxE (Table 1). With 20 simulated disease-causing SNPs, there was a cross-replicate average of 18.2 SNPs identified by GWAS and used for constructing the overall PRS, including an average of 4.7 of those 5 SNPs simulated to have a GxE interaction. Power to detect PRSxE interaction using the overall PRS ranged between 41% and 45% across multiple scenarios. When the 5 SNPs simulated to have a GxE effect were synonymous with the 5 SNPs in the pathway, power of the pPRSxE test was substantially higher (90%, scenario 1). This demonstrates the increased efficiency in focusing on a well-chosen subset of SNPs and corresponding pPRSxE test rather than attenuating the interaction signal in an overall PRSxE test.

**Table 1:** Power to detect polygenic risk score by E interactions.

	# Pathway	Pathway-SNP Effects on D			Power		
Sim	SNPs	GxE - D	G - D only	No effect	PRS x E	pPRS x E	npPRS x E
1	5	5	0	0	44%	90%	2%
2	5	4	1	0	41%	74%	4%
3	5	3	2	0	41%	47%	7%
4	5	2	3	0	45%	23%	17%
5	5	1	4	0	45%	7%	28%
6	5	4	0	1	41%	84%	4%
7	5	3	0	2	41%	69%	7%
8	5	2	0	3	45%	52%	14%
9	5	1	0	4	45%	27%	25%

Simulated power based on 1,000 replicates. Each replicate includes 15 SNPs with a G-only effect on D and 5 SNPs with a GxE effect on D. There are 5 SNPs in the pathway. Each simulation scenario varies the number of pathway SNPs that overlap with the GxE SNPs (GxE-D), G-only SNPs (G-D), and no-effect SNPs. Power is the proportion of replicates in which the null hypothesis of no interaction is rejected when the polygenic score is based on all GWAS significant SNPS (PRS x E), GWAS SNPS in the pathway (pPRS x E), or GWAS SNPS not in the pathway (npPRS x E).

We also considered simulation scenarios in which only a subset of the 5 pathway SNPs overlapped with the 5 GxE SNPs. These included scenarios in which the pathway SNPs without a GxE effect either did (Table 1, Scenarios 2–5) or did not (Scenarios 6–9) have a main (G only) effect on the trait. When the 5 pathway SNPs include 4 with true GxE and 1 G-only (scenario 2) or 3 GxE and 2 G-only (scenario 3), power of the pPRSxE test was still greater (74%, 47%, respectively) than the PRSxE test. However, with 2 GxE and 3 G-only (scenario 4) or 1 GxE and 4 G-only (scenario 5), power of the pPRSxE was lower (23%, 7%, respectively). By comparison, when the 5 pathway SNPs included 4 with true GxE and 1 with no effect on the trait (scenario 6), power was 84%, larger than the 74% when the non-GxE SNP had a G-only effect (scenario 2). This is because in scenario 6 the non-GxE SNP likely is not discovered in the initial GWAS and thus is not used in forming the pPRS (or PRS) score, and therefore is not attenuating the signal in the remaining GxE SNPs. This trend is further exemplified by the corresponding higher powers in scenarios 7, 8, and 9 compared to scenarios 3, 4, and 5, respectively.

As described in the Materials and Methods, for the results in Table 1 we assumed the SNP-specific power to detect SNP x E power was 10% and that each SNP had a minor allele frequency of 0.35. We observed similar patterns in power comparisons across these 9 simulation scenarios when the single-SNP x E power was higher on average (55%, S1 Table) and when SNP-specific minor allele frequencies (MAF) were allowed to vary (between 0.1 and 0.4, S2 Table).

### Colorectal cancer (CRC) application

The most recent and largest GWAS of CRC described a total of 204 previously identified and novel autosomal SNPs that reached genome-wide significance [16]. We investigated whether PRS and pPRS formed from these SNPs interact with use of aspirin or non-steroidal anti-inflammatory drugs (NSAIDs) use, a factor well-established to reduce CRC risk [17–19]. We used data from the Functionally Informed Gene-environment Interaction (FIGI) study, a consortium of 45 studies that includes 78,253 subjects (33,937 cases, 44,316 controls) with complete data on NSAIDs, genotypes, and covariates [19]. Adjusting for covariates, the NSAIDs main effect on CRC is OR=0.76 (95% C.I. 0.74, 0.79). Although NSAIDs is a protective factor on average, there are risks associated with regular use, such as gastrointestinal bleeding, that necessitate a precision prevention approach. This is one motivation for exploring a precision prevention approach for NSAIDs based on possible modification by genetic susceptibility.

**Fig 1:**
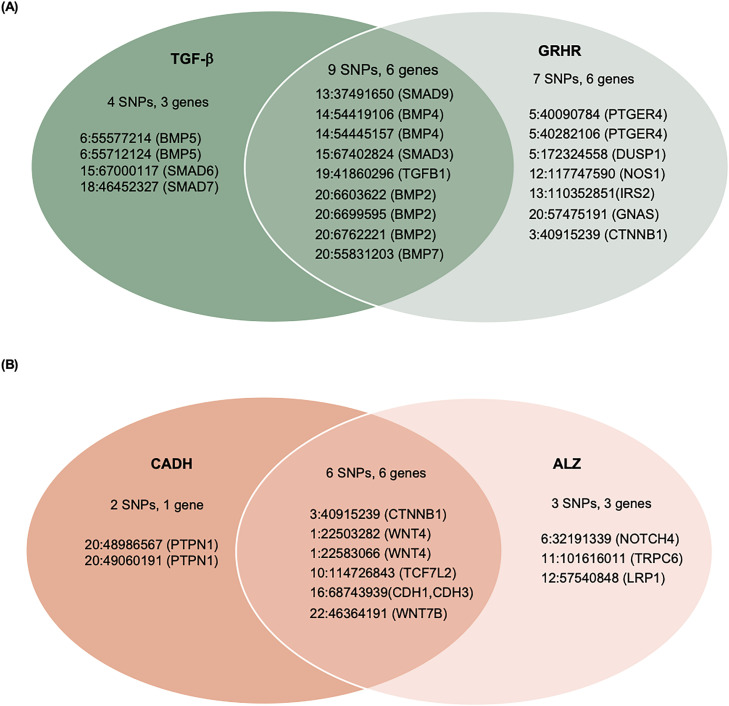
Subsets of 204 CRC-associated SNPs annotated to genes within the: (A) TGF-β and/or the Gonadotropin releasing hormone receptor (GRHR) pathways, or (B) Cadherin signaling (CADH) and/or the Alzheimer’s disease-presenilin (ALZ) pathways.

We constructed an overall PRS by first applying logistic regression within the FIGI sample to model CRC as a function of the 204 GWAS SNPs, with adjustment for study, sex, age, and three global ancestry PCs (see Materials and Methods). The SNP-specific log-odds ratios estimated from this model were used as the weights *[w]* to construct a PRS_i_, i = 1, …, N for each study subject (S3 Table). To construct pPRS, we used ANNOQ [20] to annotate SNPs to genes and PANTHER [21] to annotate genes to pathways (S4 Table). Among the identified pathways, overrepresentation analysis identified 21 with an FDR < 1.0 (Table 2), of which four included more genes than expected by chance alone at a false discovery rate (FDR) of 0.05. Additional details of the annotation process are provided in Materials and Methods. The four overrepresented pathways included the TGF-β signaling pathway, Gonadotropin-releasing hormone receptor pathway, Alzheimer disease presenilin pathway, and the Cadherin signaling pathway. A total of 30 of the 204 SNPs were annotated to genes in these pathways (Fig 1). Subsets of the above PRS weights were utilized to construct the corresponding four pPRS scores. Annotated genes in the TGF-β signaling (TGF-β) pathway and Gonadotropin-releasing hormone receptor (GRHR) pathways are highly overlapped (Fig 1A), as are genes in the Cadherin signaling (CADH) and Alzheimer’s disease presenilin(ALZ) pathways (Fig 1B). These overlaps lead to significant correlations between the computed pPRS scores for TGF-β and GRHR (R^2^ = 0.58) and for CADH and ALZ (R^2^ = 0.71). Given this, we also constructed two additional pPRS scores based on SNPs within the combined subsets of TGF-β/GRHR genes and CADH/ALZ genes, respectively.

**Table 2:** Pathways with Overrepresentation FDR < 1.0 Based on Annotation of 204 Colorectal-Cancer-associated SNPs to Genes.

PANTHER Pathways	Total # genes in pathway	# CRC* genes based on SNP-gene annotations	Expected # CRC genes by chance	Fold Enrichment	Unadjusted p-value	FDR
TGF-beta signaling pathway (P00052)	100	9	1.29	6.99	0.000006	0.0005
Gonadotropin-releasing hormone receptor pathway (P06664)	231	12	2.97	4.03	0.000048	0.0019
Alzheimer disease-presenilin pathway (P00004)	127	9	1.64	5.50	0.000040	0.0021
Cadherin signaling pathway (P00012)	163	8	2.10	3.81	0.0013	0.0406
CCKR signaling map (P06959)	173	7	2.23	3.14	0.0072	0.165
Wnt signaling pathway (P00057)	306	10	3.94	2.54	0.0065	0.174
PDGF signaling pathway (P00047)	144	6	1.85	3.24	0.011	0.220
Glycolysis (P00024)	20	2	0.26	7.77	0.027	0.392
p53 pathway feedback loops 2 (P04398)	50	3	0.64	4.66	0.027	0.425
Methionine biosynthesis (P02753)	2	1	0.03	38.83	0.026	0.455
Axon guidance mediated by Slit/Robo (P00008)	25	2	0.32	6.21	0.041	0.502
Integrin signalling pathway (P00034)	192	6	2.47	2.43	0.038	0.512
Purine metabolism (P02769)	5	1	0.06	15.53	0.063	0.717
Angiogenesis (P00005)	169	5	2.18	2.30	0.068	0.724
p53 pathway (P00059)	88	3	1.13	2.65	0.105	0.882
Endothelin signaling pathway (P00019)	87	3	1.12	2.68	0.102	0.908
Notch signaling pathway (P00045)	45	2	0.58	3.45	0.114	0.913
ATP synthesis (P02721)	8	1	0.10	9.71	0.099	0.927
Interleukin signaling pathway (P00036)	96	3	1.24	2.43	0.127	0.967
p38 MAPK pathway (P05918)	41	2	0.53	3.79	0.098	0.977
Cholesterol biosynthesis (P00014)	13	1	0.17	5.97	0.155	0.993

* CRC: Colorectal Cancer.

The estimated GxE odds ratio (OR_GxE_) for the overall PRS x NSAIDs interaction is 0.99 and is not statistically significant (p = 0.41, Table 3). We also did not observe significant pPRS x E interactions for the CADH and ALZ pathways. However, the pPRS x NSAIDs interaction was significant for both the TGF-β (OR_GxE_ = 0.96, p = 0.0069) and GRHR (OR_GxE_ = 0.96, p = 0.016) pathways. The TGF-β and GRHR pathways combined include 20 of the 204 SNPs (Fig 1A). The pPRS x NSAIDs interaction is more pronounced (OR_GxE_ = 0.94, p = 0.0003) based on the pPRS formed from this joint set of TGF-β and GRHR SNPs (Table 3). This estimate can be interpreted as an additional 0.94 protective effect of NSAIDs on CRC risk per increase of 1 standard deviation in the combined TGF-β/GRHR pPRS.

**Table 3:** Analysis of polygenic risk score x NSAIDs interaction for Colorectal Cancer.

		PRS		E (NSAIDs use)		PRS x E		
PRS Type	# SNP	OR^a^	(95% CI)	OR	(95% CI)	OR	(95% CI)	p-value^b^
PRS: All SNPs*	30	1.63	(1.61, 1.66)	0.76	(0.74, 0.79)	0.99	(0.95, 1.02)	0.41
4 Pathways^&^								
pPRS: TGF-β	13	1.18	(1.16, 1.20)	0.76	(0.74, 0.79)	**0.96**	**(0.93, 0.99)**	**0.0069**
pPRS: Gonadotropin-receptor	16	1.17	(1.15, 1.19)	0.76	(0.74, 0.79)	**0.96**	**(0.93, 0.99)**	**0.016**
pPRS: Cadherin-signaling	8	1.10	(1.09, 1.12)	0.76	(0.74, 0.79)	1.00	(0.97, 1.04)	0.82
pPRS: Alzheimer’s presenillin	9	1.09	(1.08, 1.11)	0.76	(0.74, 0.79)	0.99	(0.96, 1.02)	0.46
2 Combined Pathways^&^								
pPRS: TGF-β/Gonadotropin-receptor	20	1.21	(1.19, 1.23)	0.76	(0.74, 0.79)	**0.94**	**(0.92, 0.97)**	**0.0003**
pPRS: Cadherin/Alzheimer’s presenillin	11	1.11	(1.10, 1.13)	0.76	(0.74, 0.79)	1.00	(0.97, 1.03)	0.86
PRS Other^#^	174	1.55	(1.53, 1.58)	0.76	(0.74, 0.79)	1.01	(0.98, 1.04)	0.63

* PRS formed based on 204 GWAS significant SNPS as reported in Fernandez-Rozadilla et al. (2022).

& pPRS based on subsets of the 204 SNPs within the indicated pathway.

# PRS based on the subset of 174 of the 204 SNPs that are not within any of the indicated pathways.

a Odds ratios (OR) are scaled to a 1 s.d. increase for the indicated PRS and compare users to non-users for NSAIDs. All p < 10^–10^.

b p-value for the test of the null hypothesis of no PRS x E interaction.

**Fig 2:**
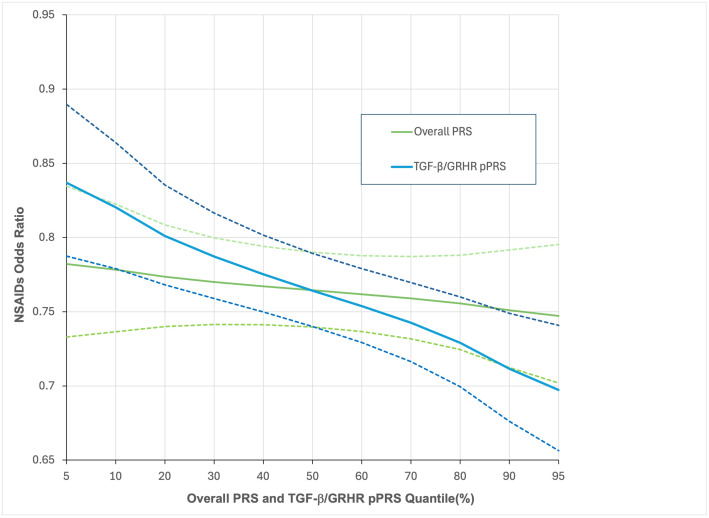
NSAIDs Odds Ratio (with 95% confidence bands) for CRC by Quantiles of the Overall PRS and TGF-β/GRHR pPRS.

To further explore and compare these results, we used the models to predict the NSAIDs effect on CRC at various percentiles of the overall PRS and TGF-β/GRHR pPRS (Fig 2). There is very little variation in the NSAIDs effect across the range of the overall PRS, which is expected given the non-significant PRS x NSAIDs interaction effect. On the other hand, the NSAIDs effect does vary substantially across the range of the TGF-β/GRHR pPRS. Specifically, for those at the 5^th^ percentile of the pPRS (low risk), the estimated NSAIDs OR is 0.84 (95% C.I. 0.79, 0.89) while at the 95^th^ percentile (high risk), it is 0.70 (0.65, 0.74). Put another way, regular NSAIDs use is predicted to reduce CRC risk by 16% for those at low risk based on the TGF-β/GRHR pPRS and by 30% for those at high TGF-β/GRHR pPRS risk.

We repeated these analyses utilizing PRS weights obtained from the PGS catalog (PGS-ID 003850) for the same set of SNPs (S3 Table). This was done to further evaluate how use of our own data to estimate PRS weights (as above) compared to the more standard approach of using catalog-derived, published weights. Applying the two sets of weights to our analysis sample yielded PRS scores that were very highly correlated for the overall PRS (R^2^ = 0.9) as well as for the TGF-β (0.98), GRHR (0.97), CADH (0.97), and ALZ (0.89) pPRS. Not surprisingly, then, results based on PGS catalog weights (Table 4) were very similar to those reported above (Table 3), with similar interaction estimates and levels of significance for TGF-β,GRHR and the joint TGF-β/GRHR pPRS x NSAIDs effects, and non-significant results for the other pathway and overall PRS x NSAIDs tests. We also performed single-SNP GxE interaction analyses for the 20 SNPs included in the joint TGF-β/GRHR pPRS (S5 Table). After Bonferroni correction for 20 tests, none of these single-SNP interactions achieved statistical significance.

**Table 4:** Analysis of PGS Catalog derived polygenic risk score x NSAIDs interaction for Colorectal Cancer.

		PRS		E (NSAIDs use)		PRS x E		
PRS Type	# SNP	OR^a^	(95% CI)	OR	(95% CI)	OR	(95% CI)	p-value^b^
PRS: All SNPs*	30	1.59	(1.56, 1.61)	0.77	(0.74, 0.79)	0.98	(0.95, 1.01)	0.24
4 Pathways^&^								
pPRS: TGF-β	13	1.18	(1.16, 1.20)	0.76	(0.74, 0.79)	**0.96**	**(0.93, 0.99)**	**0.009**
pPRS: Gonadotropin-receptor	16	1.17	(1.15, 1.18)	0.76	(0.74, 0.79)	**0.96**	**(0.94, 1.00)**	**0.021**
pPRS: Cadherin-signaling	8	1.10	(1.08, 1.11)	0.76	(0.74, 0.79)	1.00	(0.97, 1.03)	0.84
pPRS: Alzheimer’s presenillin	9	1.08	(1.07, 1.10)	0.76	(0.74, 0.79)	0.99	(0.96, 1.02)	0.64
2 combined Pathways^&^								
pPRS: TGF-β/Gonadotropin-receptor	20	1.21	(1.19, 1.23)	0.76	(0.74, 0.79)	**0.95**	**(0.92, 0.98)**	**0.0004**
pPRS: Cadherin/Alzheimer’s presenillin	11	1.10	(1.09, 1.12)	0.76	(0.74, 0.79)	1.00	(0.97, 1.03)	0.998
PRS Other^#^	174	1.51	(1.49, 1.53)	0.77	(0.74, 0.79)	1.00	(0.97, 1.03)	0.957

* PRS formed based on 204 GWAS significant SNPS as reported in Fernandez-Rozadilla et al. (2022).

& pPRS based on subsets of the 204 SNPs within the indicated pathway.

# PRS based on the subset of 174 of the 204 SNPs that are not within any of the indicated pathways

a Odds ratios (OR) are scaled to a 1 s.d. increase for the indicated PRS and compare users to non-users for NSAIDs. All p < 10^–10^.

b p-value for the test of the null hypothesis of no PRS x E interaction.

## Discussion

We have demonstrated by simulation and application to data that forming a PRS based only on a subset of GWAS significant SNPs, specifically a subset defined a priori based on pathway information, has the potential to better identify novel PRS x E interactions. We also demonstrate that power may be reduced using the standard practice of testing PRS x E interaction based only on an overall PRS. This power reduction is likely due to dilution of the interaction signal with the inclusion of most of the SNPs in the PRS construction that do not have any role in modifying the effect of E on disease. By contrast, the use of external pathway information to form a pPRS has the potential to improve power by focusing on genetic variation within a particular pathway that modifies the E effect. Examination of E effects across quantiles of the pPRS can identify those genetically-defined subsets that are most affected, or protected, by exposure. For example, our analysis of CRC suggests that although NSAIDs use is generally beneficial for all, those with the highest TGF-β/GRHR pathway PRS experience a significantly greater reduction in CRC relative risk with regular NSAIDs use. This result both adds to the overall preventive evidence for NSAIDs on CRC risk and suggests possible biological pathways that are involved in this action. Additionally, among the set of SNPs we examined, none of the single-SNP x NSAIDs tests was significant, an indication that using external pathway annotations to combine SNP information into pPRS provided increased power to detect the interaction.

Estimates of G x E interaction (and corresponding tests) can be confounded by either measured or unmeasured variables. While we adjusted for several measured covariates (Z: including age, sex, and PCs of ancestry), Keller [22] points out that GxE interaction can be confounded by GxZ and/or ExZ interactions. In sensitivity analyses of our primary findings, we considered a model that also included pairwise interactions of pPRS and NSAIDs with each of the abovementioned covariates. None of the pPRS x Z or NSAIDs x Z interactions was statistically significant. Furthermore, simultaneous adjustment for all pPRS x Z and NSAIDs x Z interactions caused less than a 1% change to our pPRS x NSAIDs estimates, making this an unlikely source of bias (S7 Table). For example, the TGF-β/GRHR pPRS x NSAIDs effect shown in Table 2 (OR=0.945) is OR=0.950 (0.6% change) with additional adjustment for pPRS x Z and NSAIDs x Z. Confounding due to unmeasured covariates (U) can also occur, if the PRS and E are correlated and there are interactions of PRS and E with U [23]. We examined correlation of each of our PRS and pPRS with NSAIDs and found no significant evidence that they were correlated. Additionally, none of the SNPs used in constructing these polygenic scores was significantly correlated with NSAIDs use. It is therefore unlikely that our pPRS x NSAIDs findings are biased due to unmeasured confounding.

The use of pPRS in interaction testing relies on external information to identify the pathways corresponding to a particular set of SNPs. In our application to CRC, we focused on the set of 204 GWAS significant SNPs. As has been previously shown, a GxE interaction typically induces a direct disease-gene (DG) association [24–27], and so requiring some level of DG association to be included in PRSxE or pPRSxE analysis is reasonable. We also chose to focus on the subset of four pathways that were overrepresented among the annotated genes of the GWAS 204 SNPs, with the goal of enriching our pPRS analyses with CRC-related genes that may be jointly involved in affecting disease risk.

For comparison to our primary analysis, we relaxed the overrepresentation condition and generated pPRS x NSAIDs results for all 50 pathways annotated by at least one of the 204 GWAS significant SNPs (S8 Table). The interaction odds ratios for the 50 pathways show the expected distribution around the null of 1.0 (S2 Fig), The QQ-plot of -log_10_(p) for the corresponding pPRS x NSAIDs tests is well calibrated for all 50 pathways (S2a Fig) as well as for the subset of 21 with FDR < 1.0 (S2b Fig) and 4 with FDR < 0.05 (S2c Fig). All three QQ-plots demonstrate enrichment of TGF-β and GRHR, the two key pathways we identified *a priori* for the focus of our primary analyses. The additional two pathways noted in S2 Fig (p38 MapK and apoptosis signaling) were not over-represented among the 204 GWAS significant SNPs (S8 Table). For additional comparison, we also relaxed the GWAS significance threshold to 1x10^-5^ and generated pPRS x NSAIDs results for the resulting 1,328 SNPs (pruned for LD), which spanned 80 pathways (S9 Table and S3 Fig). Among the 1,328 SNPs, a substantial number were annotated to the TGF-β (288) and GRHR (243) pathways, with a dilution of the corresponding pPRS x NSAIDs effect estimates compared to results based on GWAS significant SNPs (S9 Table). The QQ-plots show good calibration for all 80 pathways as well as for the subset of 7 with overrepresentation FDR < 1.0 (S4 Fig).

Taken together, these additional comparison analyses suggest that, at least in this application, a joint focus on GWAS-significant and overrepresented subsets of SNPs may be most efficient for detecting pPRS x E interactions. It is not clear, however, whether these trends would also hold for other traits and exposures, and how the sensitivity of results would depend on the number of lead variants used in identifying pathways and constructing pPRS. Whether to include additional SNPs/genes within selected pathways, additional SNPs that flank identified genes, and/or additional pathways not identified as overrepresented are important topics for analysts to consider in the analysis of pPRS x E for their particular trait of interest.

In our application to CRC, we created a workflow that utilized AnnoQ to annotate SNPs to genes that can then be analyzed in PANTHER to annotate genes to pathways. One of the strengths of the study is the comprehensive strategy we employ – integrating SnpEff, ANNOVAR, VEP, and the ENSEMBL and RefSeq databases – to ensure robust SNP-to-gene mapping. Additionally, we accounted for non-coding variants using PEREGRINE gene-enhancer link annotations. This approach allows us to capture potential regulatory effects from non-coding SNPs, reducing the risk of missing important functional variants that may influence pathway-level interactions. While we acknowledge that some regulatory variants may not be fully captured without incorporating eQTL or long-range chromatin interaction data collected directly from the study population, our multi-tool strategy minimized annotation discrepancies and increases the likelihood of accurately linking non-coding SNPs to their relevant genes. However, we recognize that alternative tools and databases, such as Reactome (*reactome.org*) or Gene Ontology (*geneontology.org*), can also be used for pathway or functional analysis, and different workflows may result in pathway assignments that do not fully overlap. A particular application of pPRS x E analysis could consider the use of multiple workflows, each using different tools and databases, to evaluate the sensitivity of findings to specific pathway definitions and corresponding SNP/gene assignments.

An ancillary finding in this paper is the demonstration that one can construct a PRS or pPRS in three different ways if the ultimate focus is a valid test of interaction. Approach #1 (Materials and Methods), i.e. to obtain existing PRS weights from the PGS catalog, is the one most often used. This has the advantages that the weights are typically estimated using a large and independent dataset, and that one can apply the weights to their data to estimate both PRS main and interactive effects. A potential disadvantage, however, is that the data used to generate the PGS weights may come from a population(s) that does not represent the sample used for PRS x E analysis. It is well known that cross-population application of PRS for main effects can lead to poor estimation, and the same will hold for analysis of PRS x E interactions. The advantage of Approach #2 is that it leverages the discovery of SNPs in a larger, independent population, but tailors the weights used in PRS construction to the specific population being studied for interaction. Of course, this is also not free of cross-population issues if the discovered SNPs in the independent population are not representative of the SNPs/genes affecting the trait in the study population. Approach #3, in which the study sample is used both to discover SNPs and estimate weights, is perhaps the cleanest from the standpoint of population heterogeneity but may suffer from reduced power to discover SNPs relative to larger independent studies. As we demonstrated in our CRC analysis, the flexibility to use alternative approaches for valid interaction testing provides the opportunity to evaluate the robustness of PRSxE and/or pPRSxE findings to the choice of PRS SNPs and weights.

In our work, we rely on the well-known independence between marginal G effects and GxE effects [27,28] to construct a robust and valid method for testing PRS x E and pPRS x E interaction. All three of the approaches described in Materials and Methods use only SNP-to-outcome weights in the construction of the PRS and pPRS. This guarantees that the downstream use of these polygenic scores for interaction testing will provide valid Type I errors, as we have confirmed via simulation studies. Some have proposed also incorporating SNPxE terms directly into the construction of a polygenic risk score [29–31]. While using SNP and SNPxE information to generate a PRS has the potential to improve predictive performance (e.g. R-squared, AUC), its use in the same dataset to examine PRS x E interaction can lead to greatly inflated Type I errors [31]. One may be able to develop a valid test that uses a PRS from an independent dataset built on both SNP and SNPxE effects, and that approach may provide increased power. However, the ability to focus on G-only PRS scores (self-generated or leveraging the many available scores in the PGS catalog), along with a robust annotation pipeline, makes our proposed approach applicable to a very wide range of traits and data structures.

Our results highlight that pPRSxE can identify pathways with functional relevance to the exposure’s putative mechanisms of action. In this case, we provide evidence that the protective effect of NSAIDs on CRC risk is modified by variation in the TGF-β and GRHR pathways. While the primary inhibitory activity of aspirin and other NSAIDs on PTGS1/2 (or COX1/2) has long been hypothesized as a central mechanism of their anticancer effects, the overall mode of action is still not yet clear. Several lines of functional evidence have supported a role for the TGF-β superfamily in mediating aspirin/NSAIDs protective effects against CRC [32], particularly in models of mismatch repair deficient CRC [33]. Long-term follow-up of the CAPP2 randomized, placebo-controlled trial conclusively demonstrated that aspirin is protective against CRC among patients with Lynch syndrome [34]. Lynch syndrome is also known as hereditary non-polyposis colon cancer and results from pathogenic variants within DNA mismatch repair genes, suggesting that NSAIDs protection may also extend to those with sporadic mismatch repair deficient tumors. TGF-β has also been demonstrated to induce *HPGD* [33], a prostaglandin-degrading enzyme with tumor suppressor activity that works as a catabolic antagonist for PTGS-2 activity [35]. Moreover, HPGD mucosal gene expression has been demonstrated to stratify individuals that may be more likely to experience a preventive benefit from aspirin use [36]. While other TGF-β superfamily members like GDF15 have been proposed as potential markers for precision prevention of CRC with NSAIDs [18], the role for bone morphogenetic proteins (BMPs) and SMAD family proteins in NSAID chemoprotection are less well established than they are for other agents, like metformin [37], or other physiologic processes, like osteogenic differentiation [38,39]. Similarly, functional evidence is limited for a specific role of Gonadotropin-receptor pathway overall in NSAIDs mechanisms of action. However, of those genes included in the pPRS score, prior evidence links NSAIDs anti-cancer activity with β-catenin (CTNNB1 [40–43]), GNAS [44], and PTGER4 [19], the extracellular receptor for PGE_2_ that is the major downstream prostanoid produced by PTGS-2. Combined, these results highlight that a pPRSxE approach may identify additional network nodes with potential functional relevance for future mechanistic interrogation.

We have shown that leveraging prior GWAS results combined with pathway information to construct subsets of SNPs in pPRS x E tests has the potential to improve power compared to SNP x E or overall PRS x E tests. An additional advantage of the pPRS x E analysis is that it may strengthen the evidence for a potential biological mechanism, via the involved pathway, by which E affects the outcome. Although we have focused on SNP subsets based on pathway information, we recognize there are other sources of information that could be used to create subsets. For example, subsets could be formed based on SNP-expression in a relevant tissue or cell type, or based on SNP associations with traits related to the trait of interest. Future research is needed to examine the robustness of pPRS x E analyses to the choice of annotation workflow, to the approach to creating subsets, and to demonstrate whether pPRS can be used to successfully identify novel gene-environment interactions for other complex traits.

## Materials and methods

### Notation and standard G × E and PRS × E analysis

Let *D*_*i*_ denote a disease indicator for subject *i*, *i* = 1, …, N, *E*_*i*_ an exposure of interest, and ***Z***_***i***_ a vector of adjustment covariates (e.g. age, sex, ancestry principal components). Assume one or more GWAS has been conducted, yielding a set ***G***=[*G*_*1*_*, G*_*2*_*, …, G*_*M*_] of trait associated SNPs, for example those with p < 5x10^-8^ for the test of SNP vs. D association. Assume further that a case-control sample has been obtained, with complete data for *D, E,* ***Z***, and ***G*** on each subject. For analysis of *G × E* interaction with a single SNP, we assume logistic regression model of the form:


$$\text{logit}\left[\text{Pr}\left(D|G,E,Z\right)\right]=\beta_{0}+\beta_{g}G+\beta_{e}E+\beta_{ge}G\times E+\beta_{z}Z$$ (1)

Here $\beta_{g}$ denotes the genetic ‘main’ effect quantifying the association between *G* and *D* when *E* = 0, $\beta_{e}$ is the corresponding environmental main effect, and $\beta_{ge}$ parameterizes the *G × E* interaction effect of primary interest. *G* is typically coded as the number of minor alleles, 0, 1, or 2 if it is measured or the corresponding expected number if imputed. In practice, we often center both *G* and *E* on their respective sample means yielding


$$\text{logit}\left[\text{Pr}\left(D|G,E,Z\right)\right]={\overset{―}{\beta }}_{0}+{\overset{―}{\beta }}_{g}(G-\overset{―}{G})+{\overset{―}{\beta }}_{e}(E-\overset{―}{E})+\beta_{ge}\left(G-\overset{―}{G}\right)\times \left(E-\overset{―}{E}\right)+\beta_{z}Z$$ (2)

Here ${\overset{―}{\beta }}_{g}$ parameterizes the *G* to *D* association at the mean of *E* and similarly for ${\overset{―}{\beta }}_{e}$. An advantage of this centering is that ${\overset{―}{\beta }}_{g}$ and ${\overset{―}{\beta }}_{e}$ approximate the ‘marginal’ effects of *G* and *E*, for example the direct effect of *G* on *D* ($\gamma_{g}$) that is obtained in a GWAS using the model:


$$\begin{array}{c} \text{logit}\left[\text{Pr}\left(D|G,\;Z\right)\right]=\gamma_{0}+\gamma_{g}G+\gamma_{z}Z \end{array}$$ (3)

For a collection of M SNPs, e.g. those previously identified as GWAS significant, the following logistic model is used to estimate all SNP effects in the context of a single joint model:


$$\begin{array}{c} \text{logit}\left[\text{Pr}\left(D|G,\;Z\right)\right]=\alpha_{0}+\sum {\mathrm{\backslash nolimits}}_{k=1}^{M}\alpha_{k}G_{k} \end{array}$$ (4)

We define the set of M weights [*w*_*k*_] to be the estimates [${\hat{\alpha }}_{k}$] from Model 4. The equation for generating a PRS for the i^th^ individual is


$$\begin{array}{c} {\text{PRS}}_{i}=\sum {\mathrm{\backslash nolimits}}_{k=1}^{M}w_{k}G_{ik} \end{array}$$ (5)

Replacing *G* in Equation 2 by the PRS yields the following model which we used to estimate and test for PRS x E interaction:


$$\text{logit}\left[\text{Pr}\left(D|G,E,Z\right)\right]={\overset{―}{\beta }}_{0}+{\overset{―}{\beta }}_{g}(PRS-\overset{―}{PRS})+{\overset{―}{\beta }}_{e}(E-\overset{―}{E})+\beta_{ge}\left(PRS-\overset{―}{PRS}\right)\times \left(E-\overset{―}{E}\right)+\beta_{z}Z$$ (6)

The test of interaction evaluates the null hypothesis H_0_: $\beta_{ge}$= 0 and can be based on a Wald, Score, or likelihood-ratio test from either model 2 (for SNPs) or model 6 (for PRS), with proper adjustment to the significance level to achieve the desired family-wise error rate.

### Overview of the pathway PRS x E analysis approach

Following are the steps of the proposed approach for conducting pPRS x E analysis, with reference to the subsequent sections that provide additional details.

1. Identify a collection of M SNPs that will be the focus for the development of the overall PRS and pathway PRS (see “Identification of PRS SNPs”)
2. Generate the PRS weights for all M SNPs (see “PRS Weights”)
3. Annotate the M SNPs to pathways (see “Pathway Annotation”)
4. Generate pPRS scores and estimates/tests of pPRS *x* E interaction (see “Pathway PRS”)

### Identification of PRS SNPs

The SNPs used to generate PRS weights are typically derived from a separate resource. For example, the PGS catalog [45] provides SNPs and weights for over 650 traits, including multiple sets for many of the traits. It is important that the weights come from independent data resources if the PRS will be used to examine direct risk effects on the disease of interest in the N subjects under study. In other words, if the weights are generated based on the N subjects under study, applying the resulting PRS to the same subjects will result in biased inference of the direct PRS effect on disease risk. However, we will demonstrate that the same dataset can be used to generate the PRS weights if the focus is on PRS x E interaction. The ability to ‘double use’ the same data to generate and apply the weights relies on the independence between the marginal genetic effects (estimated via Model 3) and the interaction effects (estimated via Model 2). This independence has been shown for tests of single SNPs [28] and is the basis for several 2-step genomewide GxE scan methods that screen on marginal G effects in Step 1 and use the information to prioritize SNPs for GxE testing in Step 2 [24,26,27,46]. We provide simulations in this paper demonstrating that this independence holds for use of the weights [*w*_*k*_] derived from Eq. 4 for downstream PRS x E interaction analysis.

### PRS weights

Given this independence, there are three Approaches one might consider for generating the [*w*_*k*_] and corresponding PRS:

1. Obtain [**w*_k_*] from prior studies based on one or more independent datasets. As noted above, these could come from the PGS catalog or a specific previous GWAS of the trait of interest. This will provide weights that can be applied to the N subjects under study for use in estimating PRS main and PRS x E interaction effects on D. One must be prepared to assume, however, that the weights generated from the previous population(s) are applicable to the current study population, which may not be reasonable if there are differences in ancestry [47].
2. Obtain M SNPs from prior GWAS but estimate [**w*_k_*] in the current sample that will be used for PRS x E analysis. Again the list of previously identified SNPs could come from the PGS catalog or a specific prior GWAS, but rather than use existing weights, model 5 is applied to the M SNPs in the current data to generate [*w*_*k*_]. The corresponding PRSi, i = 1, …, N, would not provide valid estimates of the PRS main effect but are valid for estimating and testing PRS x E effects. An advantage of this approach is that the weights are computed based on the demographic (e.g. sex, age, ancestry) composition of the current study. The discovery of the set of M SNPs, however, may have been based on different populations with different exposure histories and thus may not fully represent the genetic and GxE contributions in the current sample.
3. Conduct a GWAS on the current sample to both identify M SNPs and compute corresponding [**w*_k_*]. Compared to approaches 1 and 2, this has the advantage that both the selection of M SNPs and calculation of weights reflect the population structure and exposure characteristics of the current sample. On the other hand, the current sample may be smaller than prior studies and thus have less power to identify important SNPs in the GWAS discovery step.

We will demonstrate the third approach in our simulation and the first two approaches in our application to colorectal cancer.

### Pathway annotation

Human genes and their products typically function together within biological pathways to maintain proper cellular functions. SNPs located within or near gene regions have the potential to influence the pathways in which these genes are involved. We assume that the collection of M SNPs used to form the PRS include subsets of SNPs falling within different biological pathways. To assign each SNP to a pathway, we first use the Annotation Query (AnnoQ) platform [20] to derive annotations to Ensembl [48] and RefSeq [49] genes using inferences from ANNOVAR [50], SnpEff [51] and VEP [52]. SNPs residing in enhancer regions were linked to their target genes via PEREGRINE [53]. The resulting genes were annotated to pathways using the PANTHER [21] Classification System (v.18.0) [54]. Detailed SNP-gene and gene-pathway annotation information is provided in S4 Table. The set of genes falling within the same pathway were tested for overrepresentation relative to the PANTHER Pathway annotation sets [55]. Each pathway that is significantly over-represented is the focus of pPRS computation and pPRS x E interaction testing. Additional details on our annotation pipeline, along code and a worked example can be found on our Github repository (https://github.com/USCbiostats/SNP-to-Overrepresentation).

### Pathway PRS

Assuming that K pathways are identified by the above approach, we define pPRS_1_, pPRS_2_, …, pPRS_K_ to be PRS including only those SNPs within the corresponding pathway. We also let pPRS_0_ denote the PRS that includes the subset of M SNPs not annotated to any of the K pathways. Let S_k_, k = 0,…,K denote the subset of M SNPs included in the k^th^ subset. The pPRS for pathway k is then defined as:


$$\begin{array}{c} p{PRS}_{k}=\sum {\mathrm{\backslash nolimits}}_{j\in S_{k}}w_{j}G_{j} \end{array}$$ (7)

where weights are obtained by one of the three approaches described above. Note that this approach to computing pPRS implicitly assumes that the weights are generated from the full model of *D* that includes all M SNPs, which has the advantage that the weights are mutually adjusted for one another. To investigate a particular pPRS, Equation 6 can be modified to:


$$\begin{array}{c} \text{logit}\left[\text{Pr}\left(D|{pPRS}_{k},E,Z\right)\right]=\beta_{0}+\beta_{g}({pPRS}_{k}-{\overset{―}{pPRS}}_{k})+\beta_{e}(E-\overset{―}{E})+\beta_{ge}({pPRS}_{k}-{\overset{―}{pPRS}}_{k})\times (E-\overset{―}{E})+\beta_{z}Z \end{array}$$ (8)

Alternatively, one can also use a model that includes all pPRS, with form:


$$\begin{array}{c} \text{logit}\left[\text{Pr}\left(D|{pPRS}_{k},E,Z\right)\right]=\beta_{0}+\beta_{e}(E-\overset{―}{E})+\beta_{z}Z+\sum_{k=0}^{K}\beta_{g_{k}}{(pPRS}_{k}-{\overset{―}{pPRS}}_{k})+\beta_{{ge}_{k}}({pPRS}_{k}-{\overset{―}{pPRS}}_{k})\times (E-\overset{―}{E})) \end{array}$$ (9)

Additional interactions between pPRS and ***Z*** and/or between *E* and ***Z*** can also be included to account for potential confounding at the level of the pPRS x E effects [22]. We note that it is possible for a particular SNP to be annotated to two or more pathways. In this situation, there will be correlation between two pPRS that include the same SNP(s), which will require care in interpreting the resulting effect estimates.

### Simulation studies

We conducted simulation studies to: 1) evaluate the claim that the same dataset can be used to estimate the PRS weights [w_k_], construct a PRS, and obtain valid estimates and tests of PRS x E interaction, and 2) to compare the power of pPRS x E to PRS x E analysis.

We generate a dataset that includes 5,000 cases and 5,000 controls, with a binary exposure E and 1,000 randomly and independently generated SNPs per subject. We designate Q = 20 of the SNPs to affect disease risk, with Q_G_ having only a main G to D effect and Q_GxE_ having both a main and GxE effect. We further assume that Q_P_ = 5 of the 1,000 SNPs fall within a particular pathway and that Q_PG_ of the pathway SNPs have only main effect and Q_PGxE_ have a GxE effect. We vary Q_PG_ and Q_PGxE_ across simulation scenarios. For each simulation scenario, we generate 1,000 replicate datasets and use these to evaluate Type I error and power. In our first simulation, we generate each G as a binary variable with 35% population prevalence and E as binary with population prevalence 50%. Conditional on simulated G and E, disease status for each subject was generated according to a random Bernoulli distribution with probability of disease (P_D_) given by:


$$\begin{array}{c} P_{D}={\text{expit}(\delta }_{0}+\delta_{E}E+\sum {\mathrm{\backslash nolimits}}_{k\in Q_{G}}\delta_{G_{k}}G_{k}+\sum {\mathrm{\backslash nolimits}}_{k\in Q_{G\times E}}\delta_{{GxE}_{k}}G_{k}\times E) \end{array}$$ (10)

The values of [$\delta_{G_{k}}$] were determined using Quanto [56] to achieve an expected power of at least 90% to detect each of the Q SNPs in a GWAS with adjustment for 1,000 tests. The [$\delta_{{GxE}_{k}}$] values were set to achieve approximately 10% power to detect GxE interaction for each of the Q_GxE_ SNPs, assuming 20 SNPs are evaluated for SNP x E interaction post-GWAS.

For each simulated dataset, we conducted a GWAS of the 1,000 SNPs to identify the M that were significant at the 0.05/1,000 = 5 × 10^-5^ level. These M SNPs were used in a model of the form in Equation 4 to generate weights [w_k_]. We computed the standard PRS based on these M weights using Equation 5, the pathway PRS (pPRS) based on Equation 7 for the subset of M within Q_P_, and the non-pathway PRS (npPRS) based on Equation 7 for the subset of M not within Q_P_. Each simulation scenario was replicated 1,000 times and we tallied the proportion of replicates in which the null hypothesis of no interaction was rejected for likelihood ratio tests of PRSxE, pPRSxE, and npPRSxE based on Equation 8. This proportion estimated Type 1 error in simulations with Q_GxE_ = 0 and power when Q_GxE_ > 0.

Our first set of simulations shows that use of the same data set to run a GWAS, generate PRS weights, and test PRS x E interaction (approach #3, see above) preserves the desired Type I error rate for the interaction test (S6 Table). We simulate 20 disease-causing SNPs ($\delta_{G_{k}}\neq 0$ for $k\in Q_{G}$) and set $\delta_{G_{k}\times E}=0$, for all k (Eq. 11). We tested five methods to identify the SNPs to generate PRS weights: 1) Identify the M SNPs that were significant at the 0.05/1,000 = 5 × 10^-5^ level; 2) identify the M that were significant at the 0.05/10 = 5 × 10^-3^ level; 3) identify the M that were significant at the 0.05 level; 4) include the 20 disease-causing SNPs; and 5) randomly select 10 of the 20 disease-causing SNPs and 10 from the 980 null SNPs. Across all these scenarios, the estimated Type I error rate was within simulation variability of the desired 0.05 level. Since approaches #1 and #2 for generating PRS (see above) are subsets of approach #3, we conclude that their corresponding Type I error rates for PRSxE testing are also preserved.

### Data application: Colorectal cancer

We compare the above approaches in an analysis of GxE interactions for colorectal cancer (CRC). We use case-control data from an existing large consortium, the Functionally Informed Gene-environment Interaction (FIGI) study. FIGI includes 108,649 subjects (51,350 CRC cases and 57,299 controls) drawn from 45 contributing studies. No new contact of participants occurred as part of this paper. We focus on E = regular use of aspirin/NSAIDs (denoted NSAIDs from hereon), an exposure that has been repeatedly shown to reduce the risk of CRC [17–19]. A total of 78,253 subjects (33,937 cases, 44,316 controls) have complete data on NSAIDs use and are included in the analyses. Additional details of the study sample and definition of exposure are provided in Drew et al. [19].

The most recent and largest GWAS of CRC identified 204 SNPs that reached genomewide significance [16]. We apply the approaches described above to assess evidence that the PRS constructed from these SNPs interacts with NSAIDs to affect CRC risk. The overall PRS was constructed by first applying logistic regression within the FIGI sample to the 204 GWAS SNPs, with adjustment for study, sex, age, and three ancestry PCs (approach #2 described above). The log-odds ratios (“betas”) estimated from this model were used as the weights [w] to construct a PRS_i_, i = 1, …, N for each study subject.

To construct pPRS, we first used AnnoQ which successfully annotated 189 of the 204 SNPs to 265 protein-coding genes (S4 Table). The remaining 15 SNPs were mapped to non-coding genes and are ignored in this analysis. Application of PANTHER annotated 66 of the 265 genes to a total of 50 pathways, with pathways for the remaining 199 genes not identified. Among the 50 pathways, four of them included more genes than expected by chance alone at a false discovery rate (FDR) of 5%, identified by a Fisher’s Exact test in PANTHER (Table 2). These included the TGF-β signaling pathway (p = 6.0x10^-6^, FDR = 0.0005), Alzheimer disease presenilin pathway (p = 4.8x10^-5^, FDR = 0.0019), Gonadotropin-releasing hormone receptor pathway (p = 4.0x10^-5^, FDR = 0.0021), and Cadherin signaling pathway (p = 1.3x10^-3^, FDR = 0.04). A total of 30 of the 204 SNPs were annotated to genes in these pathways. Subsets of the above PRS weights were utilized to construct the corresponding four pPRS scores.

The genes annotated to the TGF-β signaling (TGF-β) pathway and Gonadotropin-releasing hormone receptor (GRHR) pathways are highly overlapped, as are genes in the Cadherin signaling (CADH) and Alzheimer’s disease presenilin(ALZ) pathways (Fig 1). These overlaps lead to significant correlations between the computed pPRS scores for TGF-β and GRHR (R^2^ = 0.58) and for CADH and ALZ (R^2^ = 0.71). Given these overlaps, we also constructed two additional pPRS scores based on SNPs within the combined subsets of TGF-β/GRHR genes and CADH/ALZ genes. Logistic regression was used to estimate and test pPRS x NSAIDs interactions for each of the pPRS scores, with adjustment for study, sex, age, and three principal components of ancestry. For each pPRS x E test, we report p-values unadjusted for multiple comparisons, with the rationale that each pathway-based PRS was constructed in advance using auxiliary information.

## Supporting information

S1 Table Power to detect PRS × E and pPRS × E interaction: Strong interactions. (XLSX)

S2 Table Power to detect PRS × E and pPRS × E interaction: Varying SNP minor allele frequencies. (XLSX)

S3 Table Weights for 204 colorectal-cancer-associated SNPs used to construct PRS. (XLSX)

S4 Table Gene and pathway annotations for 204 colorectal-cancer-associated SNPs. (XLSX)

S5 Table SNP x NSAIDs interaction results for 20 SNPs in the TGF-β/GRHR pathway. (XLSX)

S6 Table Estimated Type I error for testing PRS x E interaction based on simulation studies. (XLSX)

S7 Table Sensitivity of the pPRS x NSAIDs results to additional adjustment for 2-way interactions of pPRS and NSAIDs with model covariates. (XLSX)

S8 Table Analysis of pPRS x NSAIDs interaction for Colorectal Cancer, all pathway annotations derived from 204 GWAS significant SNPs. (XLSX)

S9 Table Comparison of pPRS x NSAIDS odds ratios for pPRS based on SNPs that were GWAS significant at the 5E-8 or 1E-5 threshold. (XLSX)

S1 Fig Distribution of pPRS x NSAIDs effects across 50 pathways annotated from 204 SNPs GWAS significant at 5E-8. (TIF)

S2 Fig QQ plots for pPRS x NSAIDs p-values for 50 pathways annotated from 204 SNPs GWAS significant at 5E-8. (TIF)

S3 Fig Distribution of pPRS x NSAIDs effects across 80 pathways annotated from 1,328 SNPs GWAS significant at 1E-5. (TIF)

S4 Fig QQ plots for pPRS x NSAIDs p-values for 80 pathways annotated from 1,328 SNPs GWAS significant at 5E-8. (TIF)
