# Supplementary figures and images for: Pathway polygenic risk scores (pPRS) for the analysis of gene-environment interaction
Source: PLoS Genet. 2025 Aug 5;21(8):e1011543. doi: 10.1371/journal.pgen.1011543 (PMC12352875; doi:10.1371/journal.pgen.1011543)

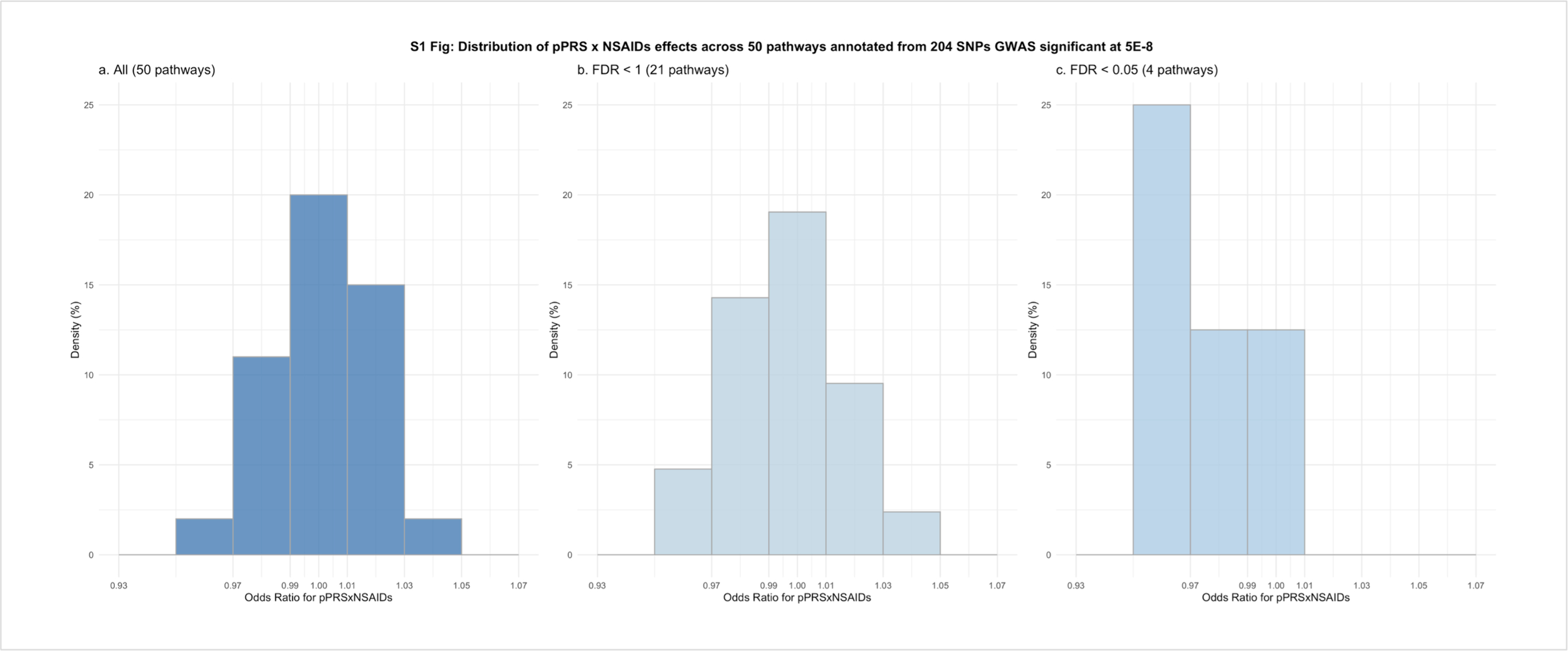

Supplement: S1 Fig — (TIF) [file pgen.1011543.s010.tif]

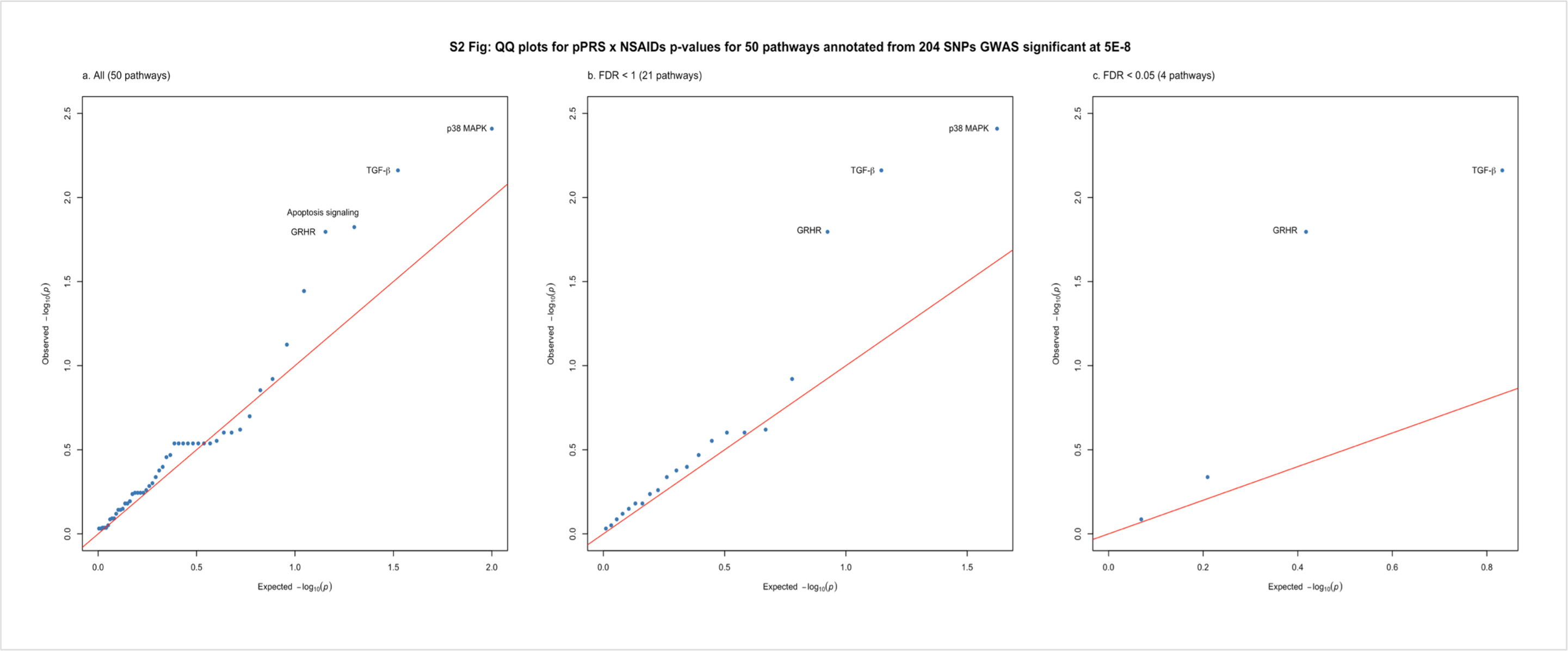

Supplement: S2 Fig — (TIF) [file pgen.1011543.s011.tif]

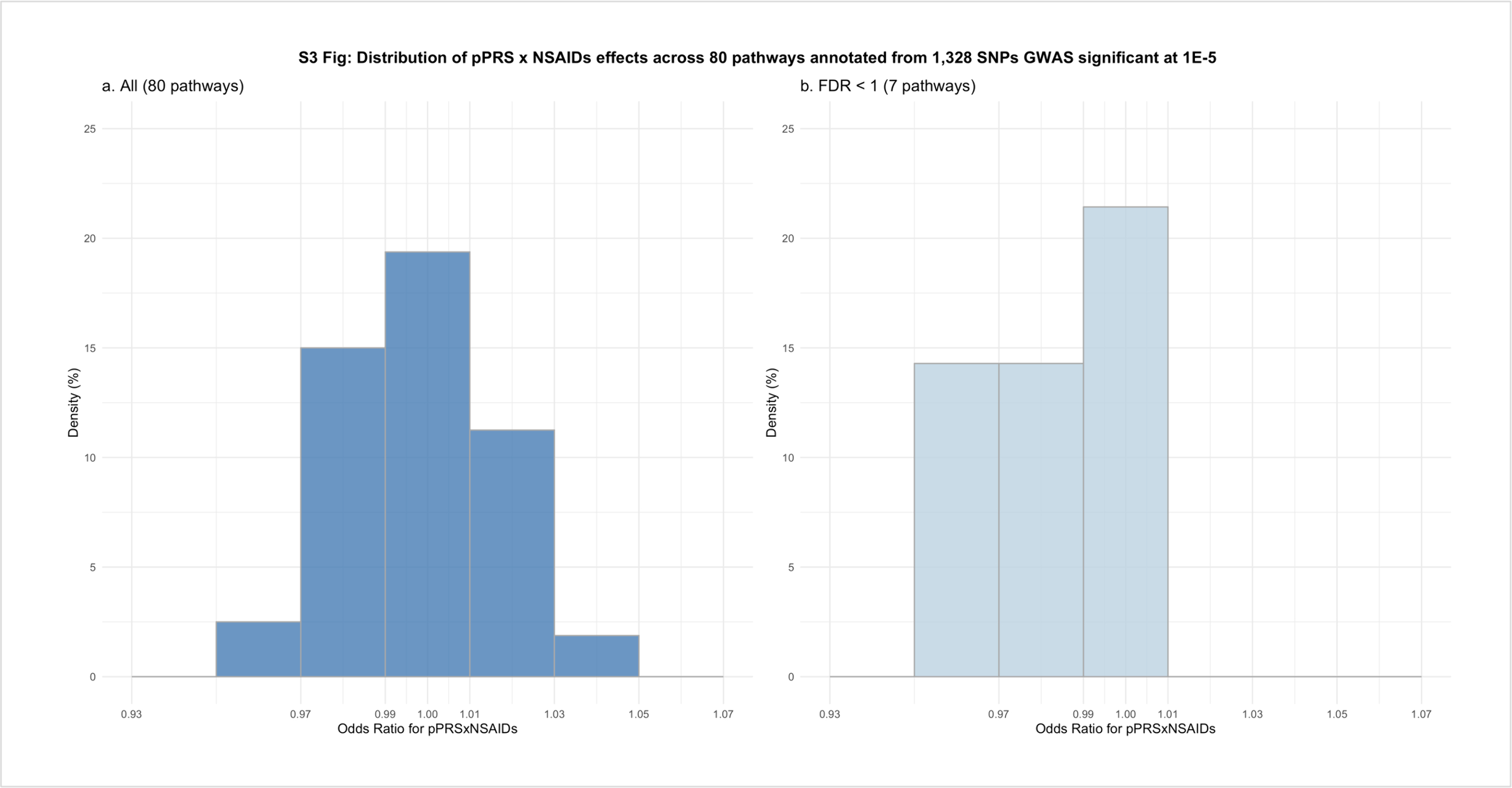

Supplement: S3 Fig — (TIF) [file pgen.1011543.s012.tif]

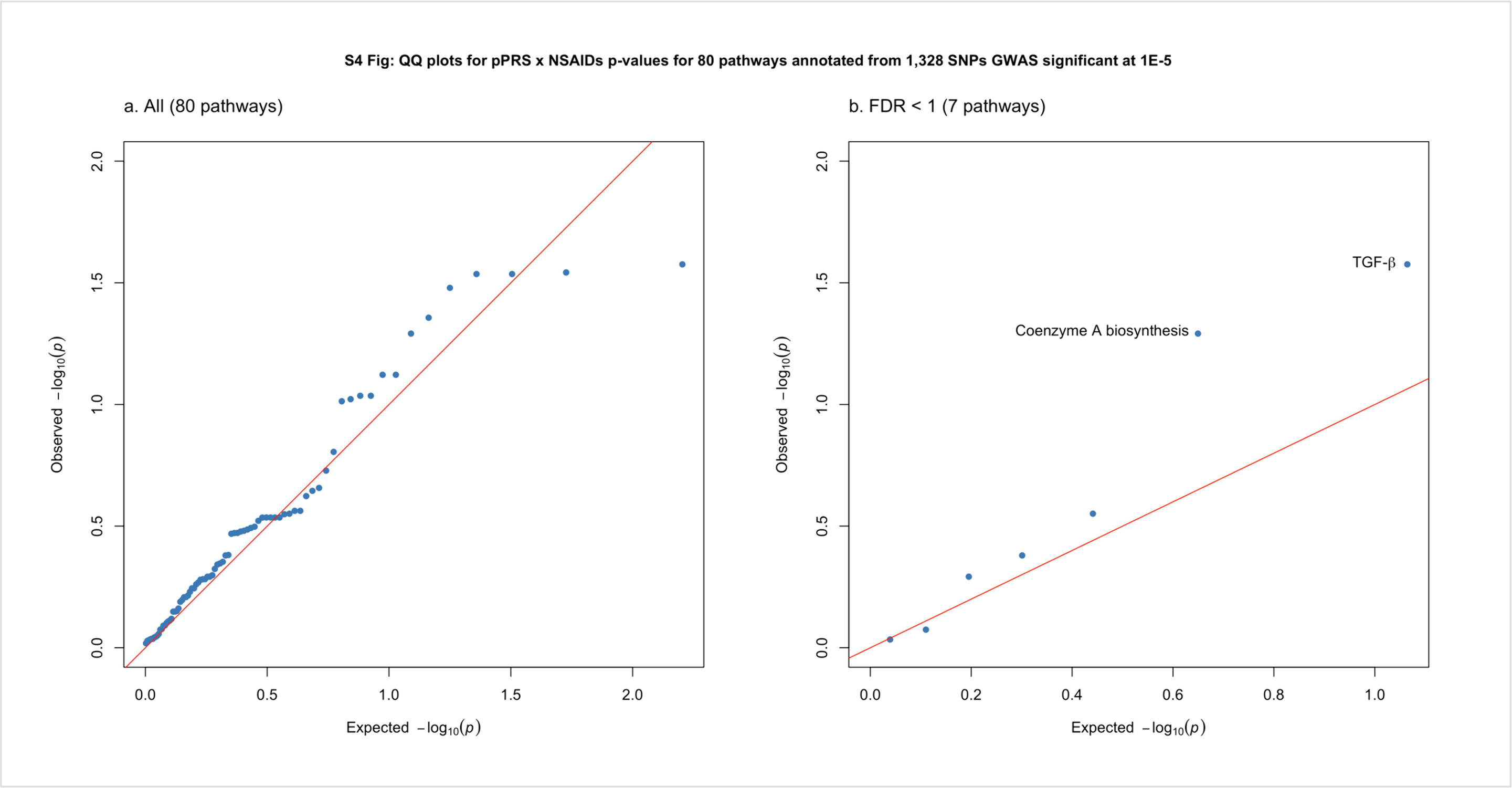

Supplement: S4 Fig — (TIF) [file pgen.1011543.s013.tif]
